# Experimental Study of Split Grouting Reinforcement Mechanism in Filling Medium and Effect Evaluation

**DOI:** 10.3390/s20113088

**Published:** 2020-05-29

**Authors:** Jiandong Niu, Zewei Li, Weiheng Gu, Kang Chen

**Affiliations:** School of Civil Engineering, Central South University, Changsha 410075, China; lizewei@csu.edu.cn (Z.L.); w15634914662@163.com (W.G.); 184812184@csu.edu.cn (K.C.)

**Keywords:** effect evaluation, experiment, filling, grouting veins, mechanism, reinforcement, split grouting

## Abstract

In view of the deficiency of the split grouting theory for the filling area, a 3D simulated grouting test system was designed to explore the slurry diffusion law, reinforcement mechanism of split grouting in a filling soil, and effect of grouting reinforcement. The test system included an experiment bench system, grouting system, and information monitoring system, using which experimental research on split grouting in a filling soil was conducted. The grouting model experiment procedure was introduced first, following which the diffusion rule of slurry in the filling medium and the reinforcement mechanism of split grouting were analyzed according to the properties and distribution characteristics of grouting veins after grouting reinforcement. Finally, a uniaxial compression test, light dynamic contact test, permeability test, and laboratory geotechnical test were conducted on the soil before and after grouting. The relationship between the zoning characteristics of different properties of veins and the mechanical properties of filling soil were discussed. The results showed that there were three types of grouting veins: trunk grouting, branch grouting, and permeable grouting. The injected soil body was strengthened by the three-stage grouting vein network of the mentioned vein types and the compaction between soils. After the grouting, the uniaxial compressive strength of the filling soil increased by an average of 186%, and the permeability coefficient decreased by an average of 47 times. The cohesion and internal friction angle increased by 45.3% and 44.9%, respectively. Additionally, density, water content, and other indicators of filling were improved. The bearing characteristics reflected by a dynamic contact test were consistent with the distribution of grouting veins. The research results offer significant guidance for the reinforcement mechanism of split grouting and the evaluation of the grouting effect.

## 1. Introduction

Grouting reinforcement technology manipulates slurry penetration, compaction, and splitting into rock cracks or soil layers. Soil with cracks and a loose structure is consolidated, forming a certain strength or impermeability of the “stone body” [1]. Grouting is a commonly used technology in infrastructure engineering. In recent years, grouting has been widely applied in underground engineering [2]. However, as grouting projects are concealed by soil, the diffusion law and reinforcement mechanism of the slurry after entering the rock–soil body cannot be directly observed [3]. In addition, different stratum conditions have different impacts on the grouting reinforcement mechanism and grouting effect. Although some scholars have carried out research on seepage grouting and splitting grouting, overall, the theoretical research on grouting engineering lags far behind engineering practice. In particular, there is a lack of systematic experimentation and research in the field of filling soil grouting; therefore, experience is often needed in grouting design, construction, and the evaluation of grouting effects, presenting an urgent problem to be solved [4,5].

At present, many scholars have done substantial research on grouting reinforcement mechanisms [6]. In terms of theoretical research [7], Yang and Hou et al. [8,9] established the rheological equation and the seepage motion equation of the time-varying viscosity of Bingham slurry, deduced the column-hemispherical infiltration grouting mechanism, and discovered the relationship between the diffusion radius of the hemispherical body and the diffusion length of the cylinder. However, they only studied the diffusion mechanism of seepage grouting; therefore, they did not provide the diffusion model of splitting grouting. Ou et al. [10] discussed the splitting grouting mechanism of pressure stabilization and pulsation. They separately deduced the slurry diffusion equation of Bingham body under two grouting methods and analyzed the relationship between grouting pressure, slurry viscosity, soil elastic modulus, split channel width, pulsating grouting frequency, and slurry diffusion range. They simplified and assumed the corresponding fracture grouting model, but only through theoretical derivation; these theories lacked corresponding experimental verification. Zhang et al. [11] simplified the splitting grouting diffusion process into a plane radiation circle based on the coupling effect of interface stress between slurry and soil. They also developed the spatial distribution equation of the width of the splitting grouting channel and the grouting pressure and analyzed the influence of slurry viscosity and the soil elastic modulus on the grouting diffusion process. It was determined that the slurry diffusion radius was negatively correlated with slurry viscosity and the soil elastic modulus, and the reinforcement mechanism of split grouting was not discussed in depth.

In terms of experimental research, Li et al. [12] carried out constant pressure permeation grouting tests of a sand medium. Through theoretical derivation and multiple regression analysis, the filtration effect of a sand-soil medium was fully considered, and the blocking mechanism and migration law of slurry in sand-soil medium were systematically revealed. However, the mechanism and effect of grouting are closely related to the type of soil. Zhang et al. [13] carried out a clay grouting indoor model test. By observing the distribution of grouting veins, the effects of grouting pressure and the slurry water–cement ratio on the diffusion of grouting were studied. The results show that split grouting can be divided into three stages: the bubble compaction stage, the first split stage, and the subsequent split stage. The research results were similar for the three types of grouting veins observed in this study. Zhang et al. [14] developed two-dimensional visual splitting grouting simulation test equipment, studied the splitting grouting reinforcement mechanism of a water-rich sand layer, and obtained the spatial attenuation characteristics of the thicknesses of grouting veins and the approximate influence range of splitting grouting. The study did not further explore the effect of grouting reinforcement on water-rich sand layers and could not provide guidance on the grouting design and construction. Li et al. [15] carried out a segmented split grouting simulation test on a soft filling medium, obtained the spatial distribution characteristics of grouting veins, revealed the mechanical mechanism of segmented split grouting reinforcement, and established a quantitative relationship between mechanical properties and impermeability of soft fillers before and after grouting.

However, in the above studies, other scholars have only studied the slurry diffusion rules from the theoretical perspective or carried out laboratory model tests on sand or clay. Owing to the particularity of grouting engineering, the randomness and uncertainty of the grouting process was ignored when only theoretical derivation and numerical simulation were used. In addition, many assumptions were needed, so it was difficult to truly reflect the grout diffusion law and reinforcement mechanism of compaction grouting and splitting grouting [16,17]. Furthermore, the grouting reinforcement mechanism, the reinforcement effect, and the characteristics of grouting veins are closely related to the type of injected soil. Previous studies mainly focused on the study of sandy soil and cohesive soil, while laboratory tests of split grouting of filling soil were relatively rare [18].

In addition, inaccurate evaluation of the grouting effect can lead to serious safety accidents, making reasonable evaluation of the grouting effect a major problem after the grouting [19]. At present, there are mainly the following methods for evaluating the grouting effect: construction quality inspection method, technical data analysis method, field observation method, borehole coring laboratory test method, borehole coring observation method, acoustic wave test method [20], TSP(Tunnel Seismic Prediction) method, borehole seismic wave CT method, geological radar method [21], standard penetration method, and static penetration method [22]. Some of the disadvantages of these methods include the following: the inability to quantitatively determine the slurry filling situation; limited investigation of the reinforcement effect; and limited qualitative analysis of the grouting effect, which lacked quantitative evaluation criteria and comprehensive results of the evaluation of various methods [23,24]. For engineering practice, the effect of grouting reinforcement cannot be accurately evaluated by a single detection method. Although in recent years, some scholars [25] have explored the detection of grouting effects by using one or more detection methods, they have failed to make a comparison with the real situation after the slurry is injected into the soil.

In summary, to investigate the slurry diffusion law and reinforcement mechanism under the condition of split grouting in the filling area and to better evaluate the effect of grouting reinforcement, a 3D grouting model test system was developed. A model test was conducted with excavated filling under the surface of the construction site of a project in Changsha, Hunan Province, China. The distribution characteristics of slurry veins were obtained, and the diffusion law and reinforcement mechanism of grouting in filling soil were studied. Through a uniaxial compression test, light dynamic penetration test, and indoor penetration test of the reinforcement bodies in different reinforcement areas after grouting, the grouting effect of the filling soil was tested. On the basis of the weighted average method, the overall improvement of filling soil performance was obtained. This study can truly reflect the process of the split grouting of the filling medium and summarize its reinforcement mechanism and performance improvement. This study offers inspiration and reference to practitioners who wish to conduct similar experiments related to the design and construction of split grouting in the fill area.

## 2. Model Experiment System of Filling Grouting

**Figure 1:**
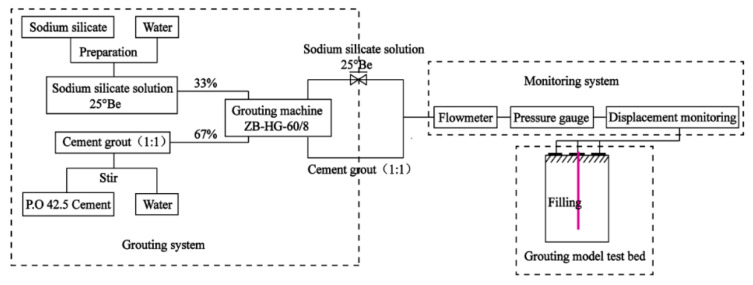
Structure diagram of the test system.

In view of the weak bearing capacity of the foundation and the poor effect of grouting reinforcement in the filling area, a 3D grouting model test system was designed to explore the slurry diffusion law and reinforcement characteristics under the condition of the split grouting of filling soil. The system can simulate the compaction grouting and splitting grouting of various soils. After the grouting, the injected medium was excavated to obtain the distribution characteristics of grouting veins, and then the slurry diffusion law and reinforcement mechanism were revealed. As the grouting process is unable to be viewed to completion in engineering applications, it is difficult to directly judge whether the grouting effect meets the needs of the project. The indoor model test can not only reflect the splitting grouting process of the filling medium, but also facilitate the sampling of the filling soil after the grouting. The in situ and indoor tests of the filled soil before and after grouting were carried out, and the test results were compared and analyzed. Combined with the characteristics of the exposed pulping veins, the improvement effect of split grouting on soil filling performance can be analyzed qualitatively and quantitatively. The structure diagram of the test system is shown in Figure 1, which mainly included the test bench system, grouting system, and information monitoring system.

### 2.1. Test Bench System

**Figure 2:**
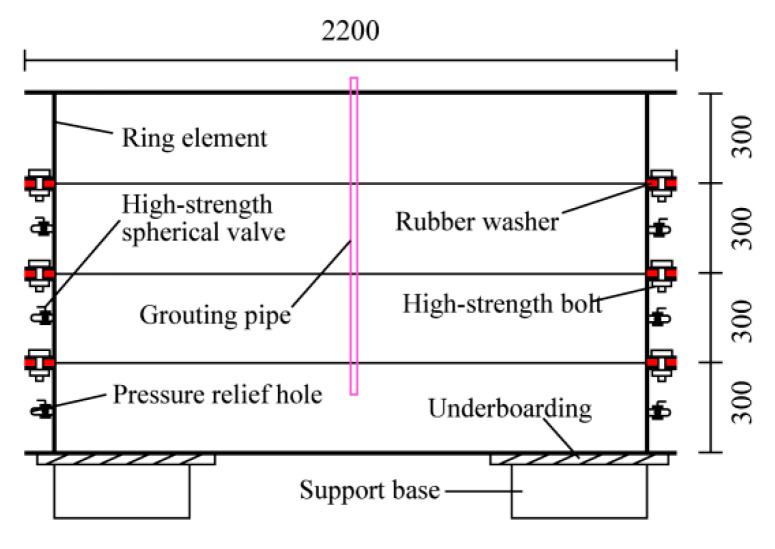
Structure diagram of model test bench.

**Figure 3:**
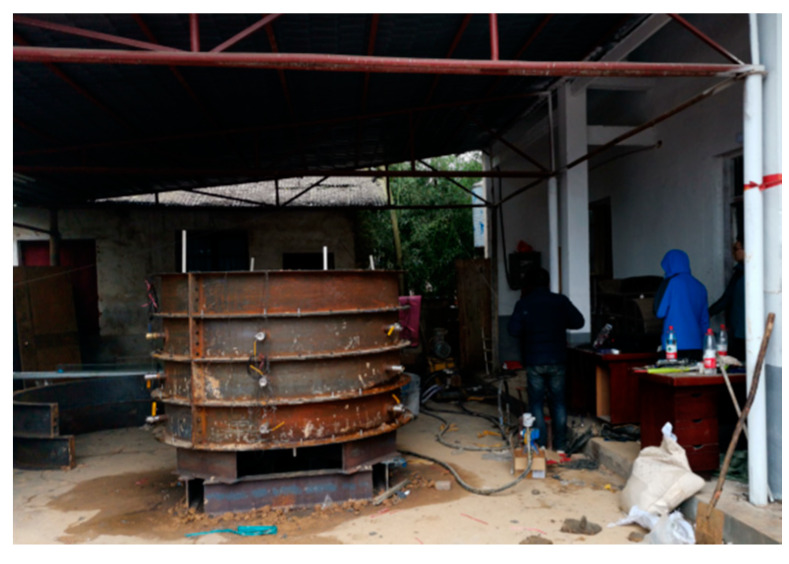
Test bench system.

To facilitate installation, disassembly, observation, and sampling, a layered combination method was used to design the model test bench. Its structural diagram and schematic diagram are shown in Figure 2 and Figure 3, respectively. The test bench consisted of eight half-rings with an inner diameter of 2 m and a height of 0.3 m and a steel plate with a thickness of 6 mm and a diameter of 2.2 m. The model cavities were made of high-strength steel with a thickness of 6 mm, and they were connected by high-strength bolts. At the connection between the layers, a rubber gasket with a thickness of 3 mm was used for sealing, and a 0.5 m high base was also provided. Six pressure relief holes were arranged in each of the three lower layers of the model cavity to prevent the leakage of slurry and fill soil during the test by means of high-strength ball valves. An electric impact tamper was used to tamp the soil. The output voltage of the tamping machine was 220 V, the impact frequency was 200–750 times/min, the take-off height was 65–75 mm, the tamping plate size was 300 × 280 mm, and the weight of the machine was 90 kg.

### 2.2. Grouting System

**Figure 4:**
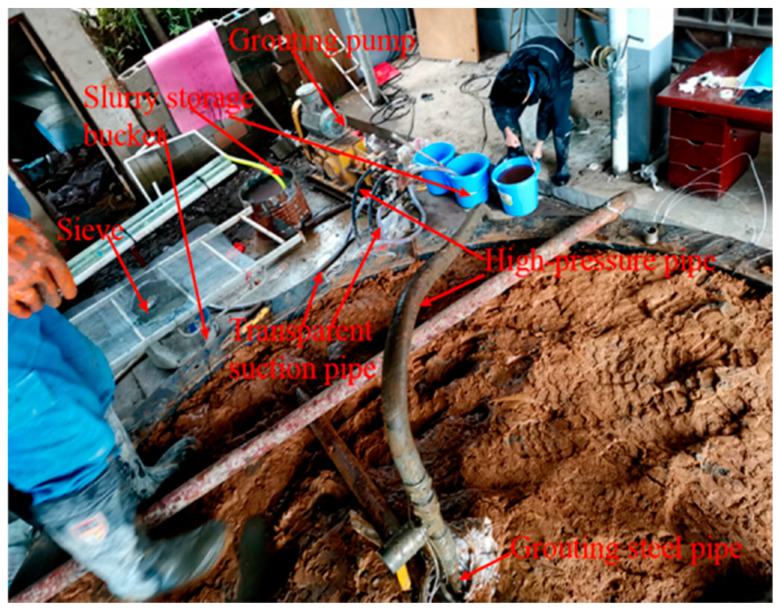
Grouting system equipment.

The grouting system mainly included the grouting pump and grouting material. The grouting pump consisted of a ZB-HG-60/8 double-fluid grouting machine with a rated flow of 60 L/min and a rated pressure of 8 MPa. The rate of grouting could be controlled by adjusting the expansion and contraction frequency of the cylinder piston. The grouting material was cement–sodium silicate (C–S) slurry commonly used in engineering. The cement was P.O42.5 ordinary Portland cement purchased from Hunan Kangda company, and the sodium silicate was ordinary sodium silicate solution from Hunan Hetang chemical company, with a modulus of 3.2 and a concentration of 25°Be. Before grouting, the cement slurry and sodium silicate solution were stored in the slurry storage bucket. In addition, the grouting system also included a mud mixer, sieve (to prevent the agglomerated cement from entering the grouting machine, which can lead to grouting efficiency decline), transparent pulp suction pipe, high-pressure pipe, and grouting steel pipe (length 1.0 m, outer diameter 25 mm, and inner diameter 20 mm), among others. Under the grouting pressure, the slurry was injected into the soil layer through the bottom of the grouting pipe, as shown in Figure 4.

### 2.3. Information Monitoring System

**Figure 5:**
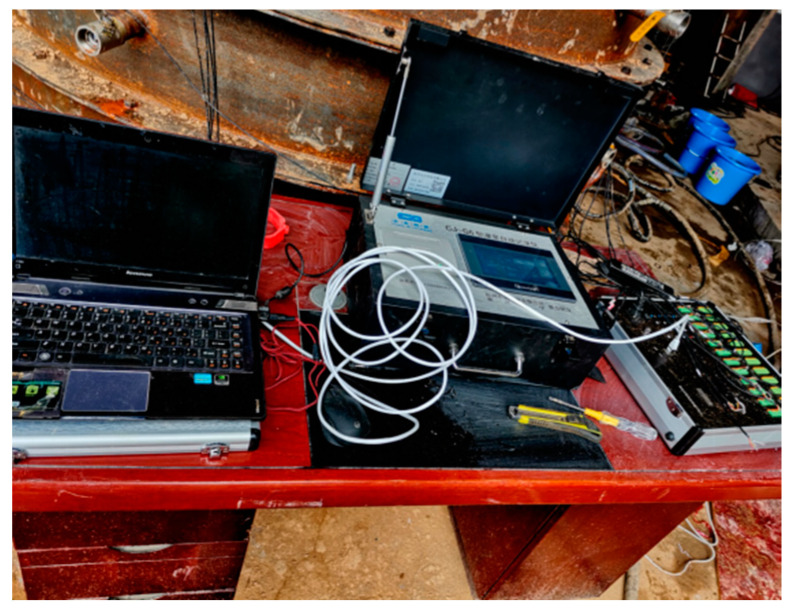
Grouting recorder.

**Figure 6:**
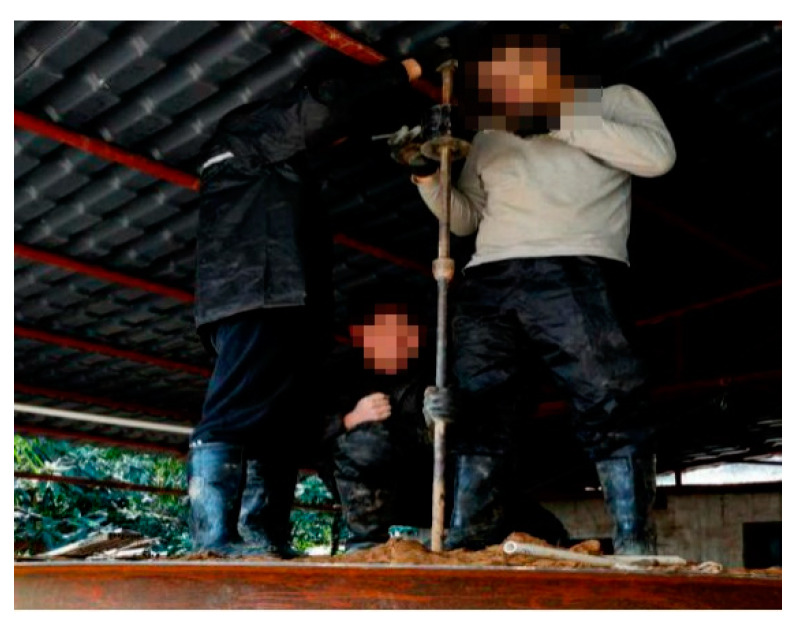
Light dynamic preliminary detector.

The monitoring system included both image acquisition and data acquisition. The data acquisition portion was composed of a CJ-G3P grouting recorder, supporting a PCM400 pressure sensor, JDK-300 electromagnetic flowmeter, and computer. The experimental results were recorded via an HD camera to provide a guarantee for the smooth progress of the test. In the experiment, the electromagnetic flowmeter was connected to the grouting line after the grouting pump in series. Both ends of the flowmeter were connected to the grouting line with matching flanges and steel pipes. The direction of the arrow on the electromagnetic flowmeter must be consistent with the direction of the slurry flow. When grouting, the grouting recorder was connected to the interface corresponding to the electromagnetic flowmeter, and the power line was connected to intervene the 220 V alternating current. During the grouting process, the grouting recorder’s pressure sensor automatically acquired the grouting pressure, flow, as well as process parameters such as density, grouting quantity, and the electrical signal. The collected data were displayed on the recorder and eventually routed to an external computer and printed out. The element of the pressure transmitter was a resistance strain gauge. The detection system was composed of a light dynamic penetrator (hammer weight 10 kg, probe diameter 40 mm, probe cone angle 60°, probe rod diameter 25 mm, and falling distance 50 mm), soil sampler, standard ring cutter, universal testing machine, and TST-55 penetrator. They were used to detect the differences in the physical and mechanical properties of the soil before and after grouting. The information monitoring equipment is shown in Figure 5 and Figure 6.

## 3. Grouting Experiment Scheme

### 3.1. Experimental Purpose

(1) The spatial distribution characteristics of grouting veins along the direction of slurry migration under the condition of split grouting in filling medium were obtained, and the reinforcement mechanism of split grouting was discussed.
(2) The physical and mechanical parameters, bearing capacity, and permeability coefficient of the injected medium before and after grouting were tested, and the improvement effect of splitting grouting on the filling strength was comprehensively evaluated.

### 3.2. Experimental Materials

The grouting material adopted the C–S double slurry commonly used in engineering. The cement is P.O42.5 ordinary Portland cement. The cement slurry water–cement ratio was m(W)/m(C) = 1:1. The sodium silicate solution used was of module 3.2 and concentration 25°Be. The volume ratio of the double slurry is V(C)/V(S) = 2:1.

**Figure 7:**
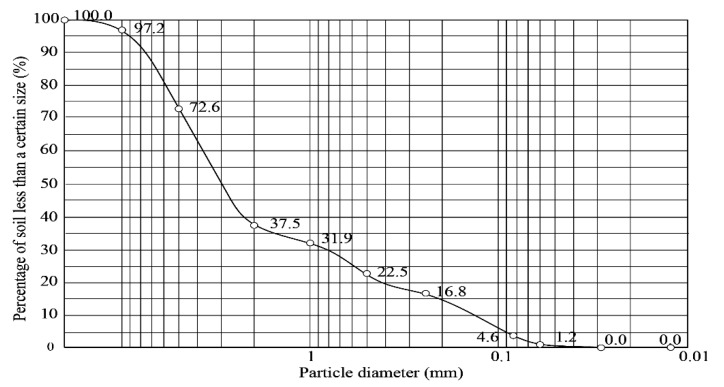
Particle size distribution curve of injected soil.

The soil used in the experiment was plain fill with a uniform texture, which was located 1–3 m below the construction site surface in the town of Datuo, Changsha city, Hunan province, China. The basic parameters of the soil are listed in Table 1, and the particle size distribution is shown in Figure 7.

The nonuniform coefficient of soil $C_{u}$ and the coefficient of curvature $C_{c}$ are expressed as follows:

(1) $$C_{u}=\frac{d_{60}}{d_{10}}=\frac{3.73}{0.15}=24.87$$

(2) $$C_{c}=\frac{d_{30}^{2}}{d_{60}\times d_{10}}=\frac{0.83^{2}}{3.73\times 0.15}=1.23$$

Both $C_{u}>5$ and $C_{c}=1\sim 3$ were satisfied at the same time, so the soil was well-graded soil.

### 3.3. Experimental Process

1. Carry out simple sieving of the filling soil retrieved from the construction site. Remove the large particles of stones, plants, and garbage; then, mix the soil to make it uniform in texture, and take soil samples at random to determine its particle size distribution.
2. Connect the pieces of equipment into a whole system as shown in Figure 1, and check its tightness. Before filling the soil, use Vaseline to smooth the inner wall of the model test cavity.
3. The filling process is carried out in layers, with the filling height of each layer not exceeding 0.3 m. After reserving a gap in the grouting pipe, the soil is shoveled into the model cavity, and the soil is evenly tamped from the center outwards with the tamping machine. Then, the soil samples of each layer are collected randomly at four locations with a standard ring cutter to test their compactness. Ensure a soil sample density difference of less than 10%. At the same time, the average density error of the soil samples in different layers should be less than 10%. If it fails to meet the requirements, the soil should be rammed again. After meeting the requirements, the next layer of soil should be filled and rammed until the four layers of soil with a total height of 1.2 m are filled.
4. Carry out light dynamic preliminary tests on the soil before grouting; the test points should be evenly and randomly distributed. Record the times that the hammer falls when the penetration instrument enters 300 mm into the soil (0–300 mm, 300–600 mm, and 600–900 mm). After the light dynamic penetration test is completed, the orifices generated during the test should be backfilled and compacted.
5. Prepare the cement slurry and sodium silicate solution. The water–cement ratio of the cement slurry should be 1:1; the same quality of water and cement should be mixed evenly through a 5 mm sieve into the slurry bucket. The sodium silicate solution is to be prepared as 25°Be and stored in another slurry bucket.
6. Conduct pre-grouting. A hole with a diameter of 70 mm and a depth of 1 m is drilled using a hydro-electric drill on the flat ground near the testbed. Lower the 1 m long grouting pipe for pre-grouting to ensure the normal use of the pressure gauge, flow meter, and grouting system. Clean the grouting pipe after the completion of pre-grouting. A 61.8 mm diameter sampler is used to sample in the center of the model barrel with a total sampling depth of 1050 mm. Then laboratory tests are carried out on the soil samples to determine their density, water content, permeability coefficient, shear strength, and compressive strength.
7. Conduct grouting. Before the grouting, drain the water from the pipeline. When thick slurry appears at the pressure relief hole, close the pressure relief hole, and then start grouting and observe the changes of the injected soil surface, slurry flow, grouting pressure, and other parameters.
8. Seven days after the grouting, a light dynamic preliminary test should be conducted again at the corresponding position.
9. Remove the shell of the test cavity, excavate the reinforced soil layer by layer from top to bottom, and observe the properties and distribution of grouting veins. Soil samples should be collected in different grouting areas, with no less than six samples in each area; then, the soil density test, water content test, permeability test, direct shear test, and uniaxial compression test should be carried out.

**Figure 8:**
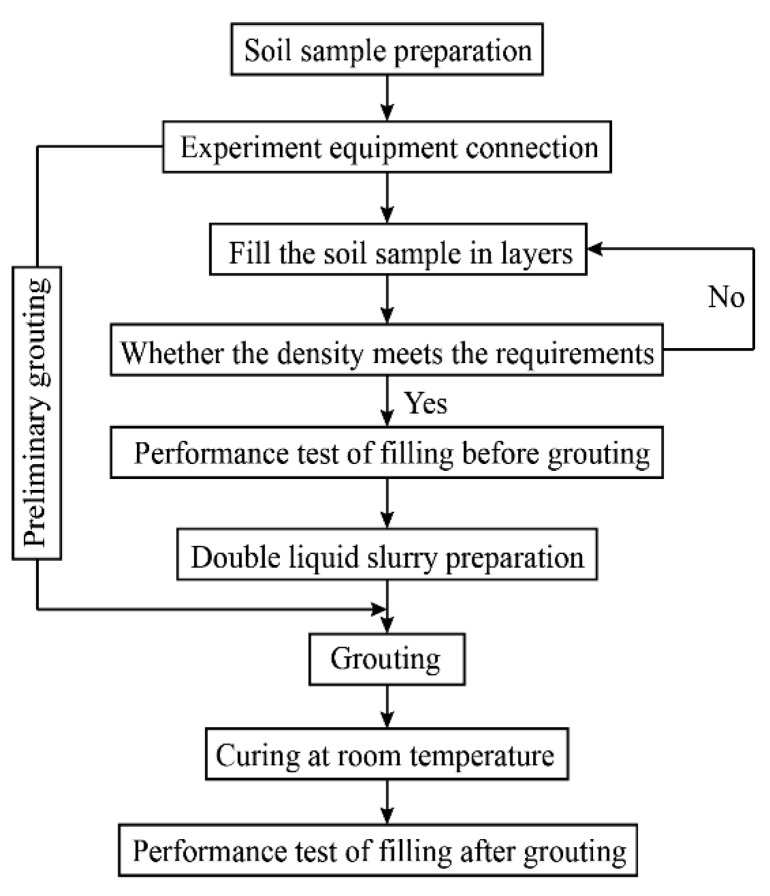
Experimental flow chart.

The experiment flow chart is shown in Figure 8.

### 3.4. Grouting Parameters

A single-pipe/single-hole grouting method was adopted in the experiment. The grouting pressure range was 0–0.5 MPa, and the grouting rate was 0–10 L/min. Take the grouting volume of 100 kg as the control standard for the end of the experiment. During the grouting process, the grouting should be stopped when the slurry appears on the top surface of the injected soil or the pressure exceeds 1 MPa.

## 4. Analysis of Experimental Results

The experimental results were analyzed from two aspects: the properties and the distribution characteristics of grouting veins and the reinforcement effect of split grouting. After the excavation of the injected soil, it was found that the slurry was mainly retained and distributed in the form of veins and slurry soil combination. The diffusion rule and reinforcement mechanism of split grouting slurry in the filling medium were studied by analyzing the properties and distribution characteristics of the slurry veins. In the evaluation of the grouting reinforcement effect, the uniaxial compression test, light dynamic touchdown test, indoor geotechnical test, and permeability test were carried out to compare and analyze the physical and mechanical properties of the injected soil before and after grouting. The improvement of the filling performance of the split grouting was obtained, and the experimental results were systematically analyzed from both qualitative and quantitative perspectives.

### 4.1. Properties and Distribution Characteristics of Grouting Veins

**Figure 9:**
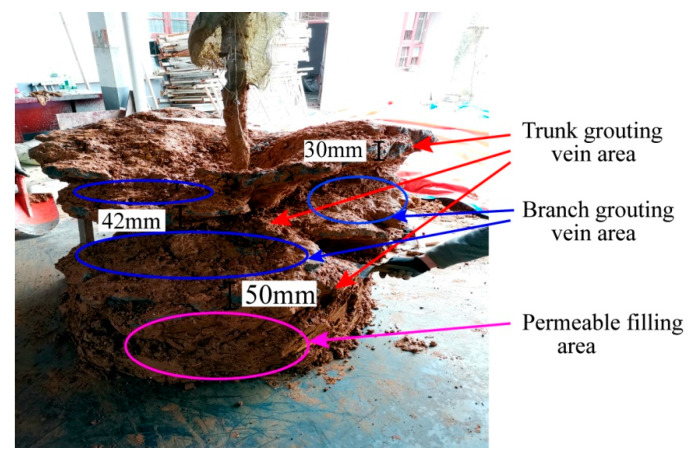
Distribution characteristics of grouting veins.

The distribution of exposed veins is shown in Figure 9. The slurry was diffused to form a semispherical slurry bubble and three trunk grouting veins. Several branch grouting veins were developed between the trunk veins, and permeable filling occurred in some areas. The injected soil was strengthened by the three-stage routing veins network of trunk vein-branch vein-infiltration diffusion.

**Figure 10:**
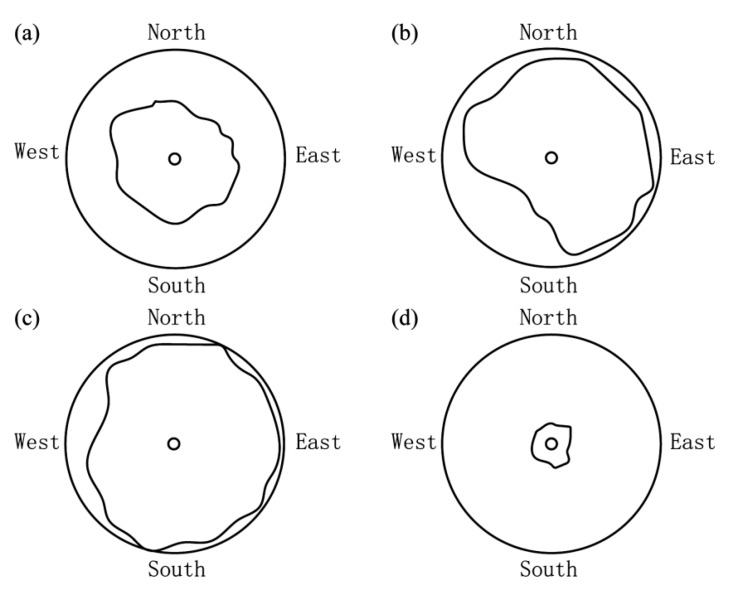
Plane distribution of trunk grouting veins. (**a**) The first trunk grouting vein, (**b**) the second trunk grouting vein, (**c**) the third trunk grouting vein, and (**d**) slurry bubble.

The trunk grouting veins were generally distributed in a horizontal plate-shape, with the grouting hole as the center. The grouting veins continued to expand further along the direction of slurry migration, forming a planar plate-shaped structure with varying thickness. The thickness of the grouting veins near the grouting hole was larger, and the thickness of the grouting veins decreased gradually as they expand further outward. In general, the trunk grouting veins of the first layer did not run through the entire filling layer, while the second and third slurry veins did run through the entire filling layer, which extended to the lateral wall of the model cavity. The planar distribution of trunk grouting veins is shown in Figure 10. In the vertical direction, the thickness of the first layer was 5–25 mm, and the thickness of the lower two layers was 20–50 mm. The reason for this may be that the grouting started, and slurry impinged downward from the orifice. Owing to the large soil pressure at the bottom and the relative compactness of the soil, as well as the vertical restraint of the bottom plate of the model cavity, the slurry could not effectively split the soil and formed the slurry bubble at the bottom layer. As the grouting pressure increased, the slurry moved up from the outside of the grouting hole and started to split the soil at other weak locations and continued to expand owing to the layered filling; therefore, the expansion speed of the upper split crack was less than that of the lower split crack. During the grouting process, the slurry first exuded from the sidewall of the model cavity, and then the slurry emerged from the top with the increase of pressure. This also verified that the slurry was mainly split horizontally in the soil layer.

**Figure 11:**
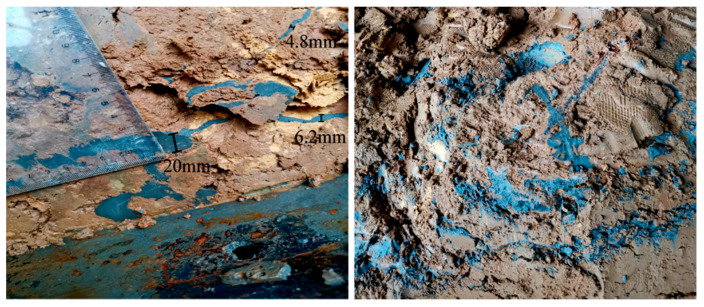
Distribution of branch grouting veins.

Between the trunk grouting veins, the intersecting branch grouting veins extend to form an intersecting network. After the trunk splitting cracks were formed, the slurry was injected into the cracks and the surrounding soil was compressed. As a result, the soil around the trunk veins was continuously compressed and its strength increased. As the pressure continued to increase, splitting would occur again on the surface of least resistance. In general, the second split plane would be perpendicular to the first split plane. With the grouting, the subsequent split plane would continue to be generated on the weak surface in the soil, and would eventually form crisscrossed branch veins that would envelop the filling structure. The branch grouting veins embedded in the filling played the role of anchorage, which was mainly distributed near the grouting hole. As shown in Figure 11, the thickness of the branch grouting veins was within the range of 2–20 mm and was less than that of the trunk grouting veins. The thickness of the branch grouting veins decreased as the distance from the grouting hole increased. Owing to the influence of the trunk grouting veins of each layer, the direction of the local branch grouting veins was irregular.

**Figure 12:**
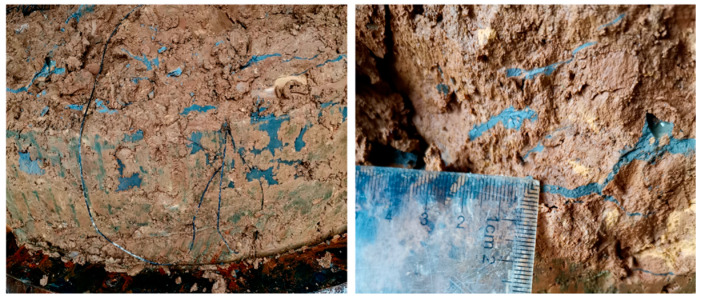
Distribution of permeable filling grouting veins.

For the permeable filling areas, the slurry penetrated the filling pores and continuously compacted the pores, so the physical and mechanical properties of the soil body were improved. The slurry appeared as irregular and discontinuous fine grouting veins, which were mainly positioned away from the grouting hole and the trunk grouting veins, near the top and bottom of the model cavity. These tiny grouting veins were less dense and lighter in color. Compared with branch grouting veins, their size and width were smaller, in the range of 0.5–2 mm, as shown in Figure 12.

### 4.2. Evaluation of Grouting Reinforcement Effect

From the characteristics and distribution of the grouting veins, the reinforcement effect of split grouting on the filling was mainly reflected in two aspects. On one hand, the trunk grouting veins replaced the filling in corresponding parts, playing the role of a supporting skeleton of the injected medium. The cross of the branch grouting veins formed a network structure, which acted as a type of reinforcement for the soil, and the branch grouting veins were embedded into the filling to play the role of anchoring. On the other hand, the slurry had significant compaction and permeation effects on the filling. The expansion of the slurry splitting channel forced the pore water pressure in the surrounding soil to rise. After grouting, the pore pressure would gradually dissipate, effective stress would increase, soil would be consolidated and compacted, and shear strength of the soil would increase accordingly.

#### 4.2.1. Uniaxial Compressive Strength

**Figure 13:**
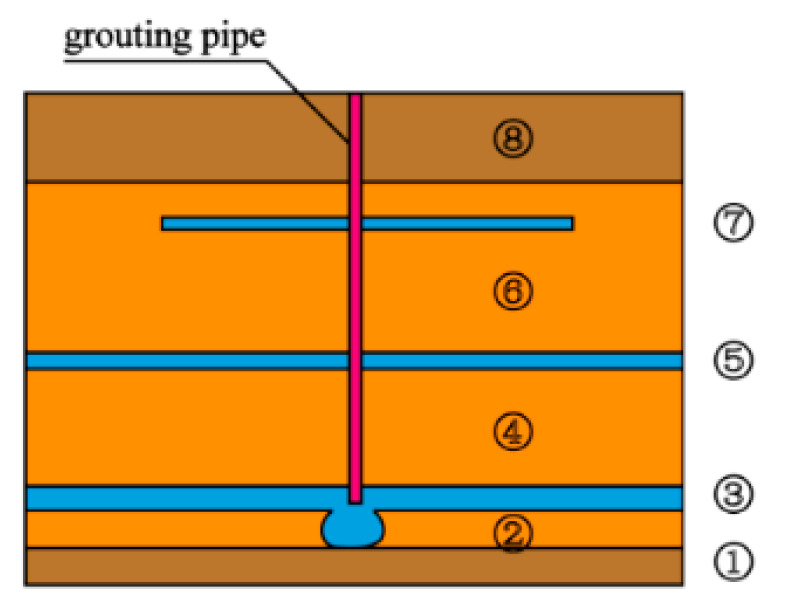
Schematic diagram of injected medium partition.

According to the distribution of grouting veins after the grouting, in order to simplify the calculation, the thickness of the trunk grouting veins was assumed to be unchanged along the expansion direction, and the areas between the trunk grouting veins were all assumed to be branch grouting veins, while the other areas were considered to be the permeable filling areas. The distribution law of the filled soil slurry veins after excavation was observed, and the map of the filled soil partition was created with the same scale, as shown in Figure 13. According to the type of pulp veins, the reinforced soil was divided into eight regions. Among them, ③, ⑤, and ⑦ were the trunk grouting vein reinforcement areas. This region was composed of three round plate-shaped regions replaced by pulps and one hemisphere region. The plate-shaped regions have the same thickness, and this region was completely filled with pulps, thus the reinforcement effect in this region was the best. Areas ②, ④, and ⑥ were the branch grouting vein reinforcement areas. Compared with the reinforcement effect in the areas of the main pulp veins, the reinforcement effect of this area was poor. It was concentrated between and around the main pulp veins within a certain thickness range, and the branch pulp veins were crisscrossed and embedded into the filling soil. The filling soil in this area was neither continuous nor completely replaced. Areas ① and ⑧ were the permeable filling reinforcement areas. It was mainly distributed in a certain thickness range at the top and bottom of the model cavity. A small part of the slurry penetrated this area to fill the soil. The slurry veins were small and dispersed, and the filling performance was improved slightly.

The compressive strength test of the filling samples was carried out, and the grouting stone body was cured for seven days at room temperature. A uniaxial compression test of the samples in each partition was conducted with the CB-WAW-1000 universal testing machine. Then, the equivalent elastic modulus of the injected soil was determined by the coefficient weighted average of the volume of each grouting reinforcement area in the whole fill volume. The uniaxial compressive strength test results of the filling samples are listed in Table 2.

The average compression modulus of the grouting reinforcement range is as follows:

(3) $$E_{i}=\frac{1}{4}\sum_{m=1}^{6}E_{i-m}$$

(4) $$E=\sum_{i=1}^{3}\frac{V_{i}}{V}E_{i}$$

where i is the area number of the injected soil, with values 1, 2, and 3, representing trunk grouting area, branch grouting vein area, and permeable filling area, respectively; m is the number of samples (range 1–6) in each area; V is the total volume of the injected soil; $V_{i}$ is the total volume of grouting vein area i; $E_{i}$ is the average elastic modulus of the vein area i; $E_{i-m}$ is the elastic modulus of the vein area i and sample m; and E is the equivalent elastic modulus of the grouting reinforcement solid.

According to Equations (3) and (4), the average compressive strength of the filling before grouting reinforcement was 6.65 MPa, and the average compressive strength of the trunk grouting vein area after grouting was 35.58 MPa; this indicates an increase of 431% after grouting reinforcement. The average compressive strength of the branch grouting vein reinforcement area was 23.18 MPa, which was 248% higher than that of the area before reinforcement. The average compressive strength of the permeable filling area was 10.7 MPa and the equivalent elastic modulus of the injected soil was 19.02 MPa, which were 61% and 186% higher than that of the area before grouting, respectively. This indicated that the three-stage grouting vein network of trunk vein-branch, vein-permeable filling veins could significantly improve the strength of the injected soil.

#### 4.2.2. Light Dynamic Preliminary Test

**Figure 14:**
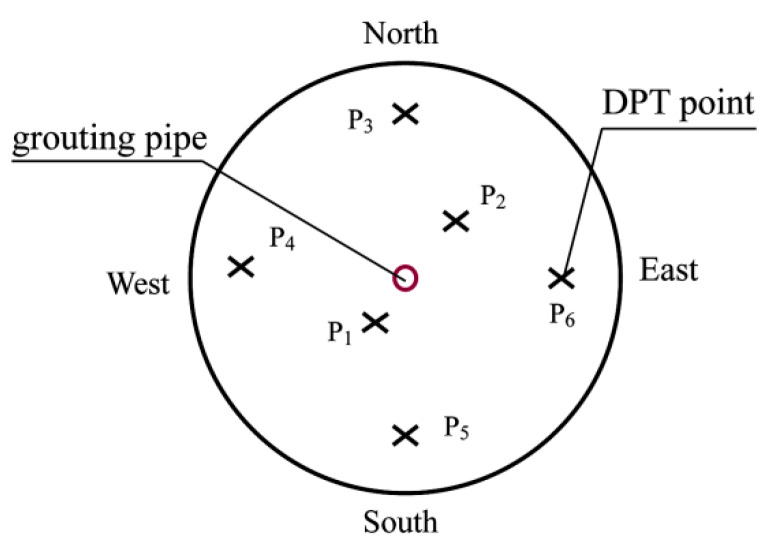
Arrangement of light dynamic penetration points.

**Figure 15:**
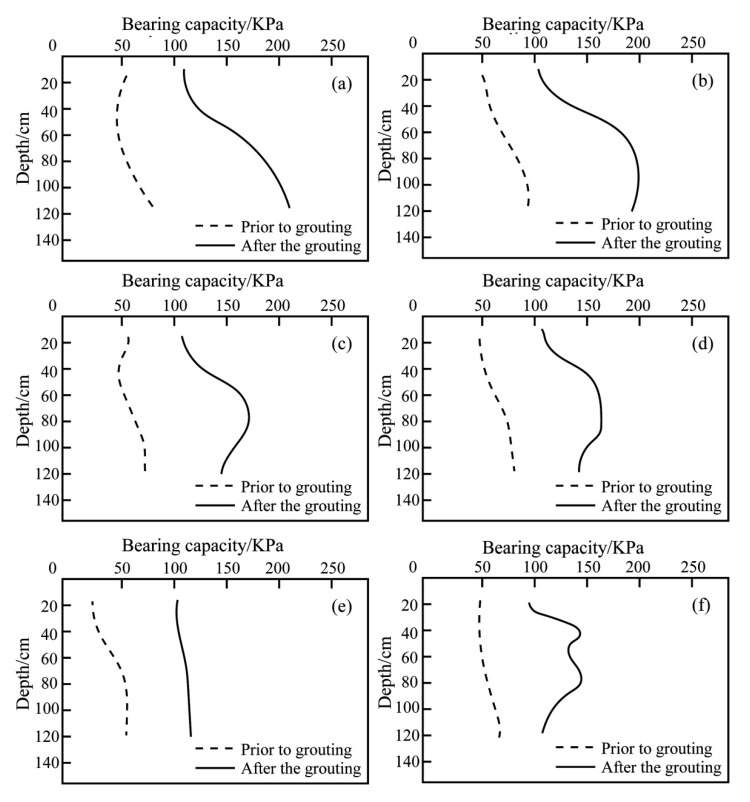
Change of bearing capacity of dynamic penetration test points. (**a**) Point 1, (**b**) point 2, (**c**) point 3, (**d**) point 4, (**e**) point 5, and (**f**) point 6.

The in situ test could well reflect the true stress–strain state of the soil under undisturbed conditions. The research used a light dynamic preliminary test to quantitatively understand the change of mechanical properties of the soil before grouting. Before and after grouting, six points were selected for the dynamic penetration test. The arrangement of penetration points is shown in Figure 14. According to the “Code for Design of Building Foundations”, the variation of bearing capacity with depth was drawn according to the number of dynamic preliminary hammer strokes, as shown in Figure 14. It can be seen in Figure 15 that the bearing capacity after grouting has been improved to different degrees compared with that before grouting. For example, point 1 and point 2 had a large increase, reaching 230 kPa and 180 kPa, respectively. This corresponded to the distribution characteristics of grouting veins. The thickness of grouting veins near the grouting hole was large, and the branch grouting veins were mainly concentrated at the grouting hole, so the bearing capacity of points 1 and 2 near the grouting hole increased greatly. Before grouting, the bearing capacity of the lower soil in the model cavity was generally larger than that of the upper part. After grouting, the bearing capacity curve showed 1–2 large peaks. The bearing capacity of the lower soil body increased more than that of the upper, which was caused by the plate-shaped distribution of trunk grouting veins and the thickness of the lower trunk grouting veins being greater than that of the upper trunk grouting veins. This showed that the split grouting was effective in lifting the bearing capacity of the foundation.

#### 4.2.3. Indoor Geotechnical Test

**Figure 16:**
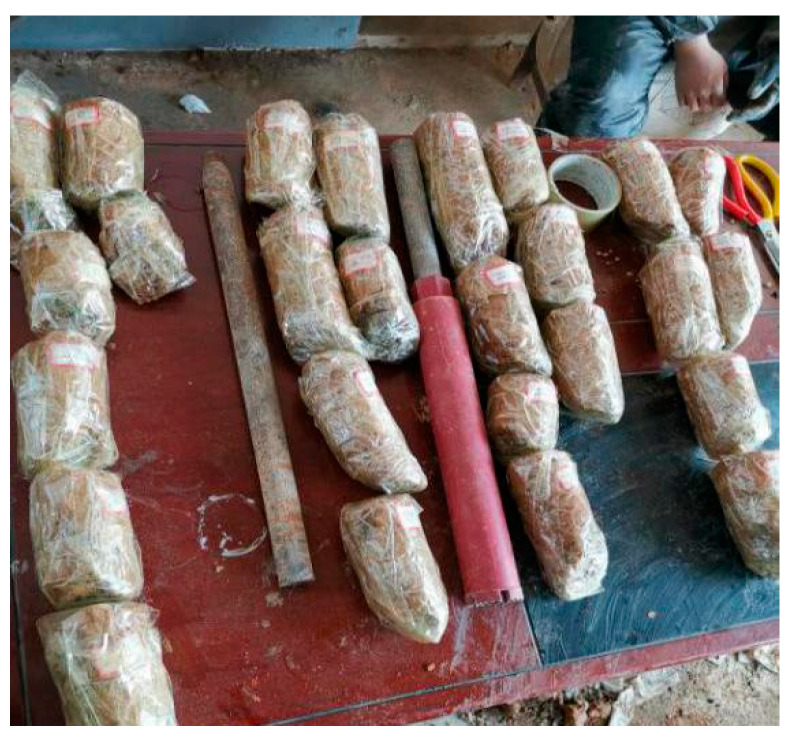
Indoor geotechnical test samples.

**Figure 17:**
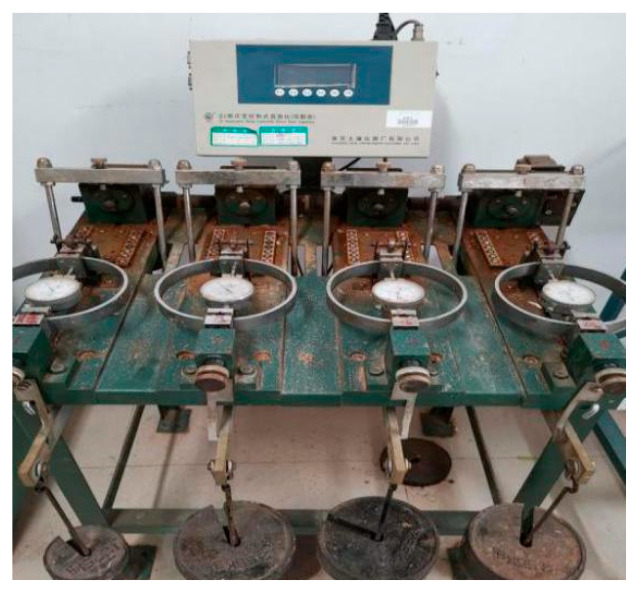
Direct shear test.

The physical and mechanical parameters of soil samples from different areas before and after grouting were measured via indoor geotechnical tests, as shown in Figure 16 and Figure 17. The test results are listed in Table 3. It could be summarized from Table 3 that, after grouting, all indexes of the filling in different areas have been improved to a certain extent, in which the average density of soil filling increased by 6.43%, and the average water content decreased by 17.93%. The cohesion and internal friction angle of the soil before grouting were 12.8 kPa and 4.9°, respectively, and the average cohesion and internal friction angle of the soil after grouting were 18.6 kPa and 7.1°, respectively. This equates to a 45.3% increase in cohesion and a 44.9% increase in internal friction angle after grouting. Among the areas, the trunk grouting vein area had the largest increase, with an average increase in cohesion and internal friction angle of 167% and 75.7%, respectively, while the soil parameter in the permeable filling area had a smaller increase. The above results indicated that grouting effectively improved the mechanical properties of loose filling.

#### 4.2.4. Permeability Test

**Figure 18:**
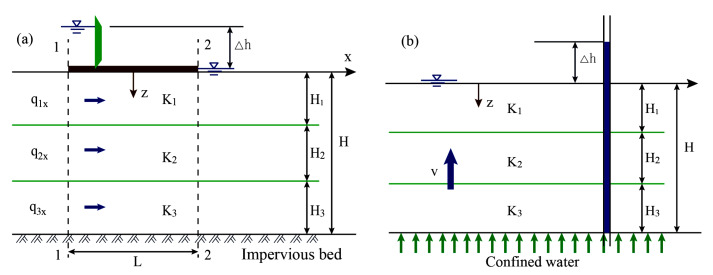
Schematic diagram of percolation in layered stratum. (**a**) Penetration coefficient in the direction of parallel planes, (**b**) Permeability coefficient in the vertical plane direction.

The permeability coefficient of soil samples was measured by a TST-55 permeameter. To avoid disturbance, a large block was cut out first during sampling; then, a cutting tool was used to cut along the edge of the ring knife. After the ring knife was embedded in the sample, the sample was truncated and leveled with the top and bottom of the ring tool as the boundary. The sample was put into the sleeve of the permeameter, and molten wax was poured into the gap between the sample and the sleeve. After the wax liquid condensed, the sample and sleeve were placed on the base for the penetration test. As the grouting veins of different characters were distributed roughly in layers in the filling after grouting, the permeability coefficient of the filling was also expected to show obvious stratification characteristics. When Darcy’s law was used to calculate the permeability coefficient of the injected soil within the grouting range, the permeability coefficient in the horizontal direction and vertical direction was calculated separately. The calculation model is shown in Figure 18.

According to the principle of seepage mechanics, the equivalent permeability coefficient of a layered foundation along the direction of parallel planes $K_{h}$ and the equivalent permeability coefficient along the direction of vertical planes $K_{v}$ were calculated as follows:

(5) $$K_{h}=\frac{1}{H}\sum_{i=1}^{n}H_{i}K_{i}$$

(6) $$K_{v}=\frac{H}{\sum_{i=1}^{n}\frac{H_{i}}{K_{i}}}$$

where $K_{h}$ is the equivalent permeability coefficient in the direction of parallel plane, $K_{v}$ is the equivalent permeability coefficient in the direction of vertical plane, H is the total thickness of the layered foundation, $H_{i}$ is the thickness of the foundation soil of layer i, and $K_{i}$ is the permeability coefficient of the foundation soil of layer i.

The equivalent permeability coefficients in the horizontal and vertical directions of the injected soil were calculated according to Equations (5) and (6), respectively. The results showed that $K_{h}>>K_{v}$, which could be attributed to the stratification of horizontal platy grouting veins and reticular branching grouting veins, restricting the migration of pore water. Therefore, the equivalent permeability coefficient in the horizontal direction was used to represent the permeability coefficient of the grouting reinforcement body. The results of the permeability coefficient in different areas after grouting are shown in Table 4. According to Table 4 and Equation (5), the equivalent permeability coefficient of the grouting reinforcement body was $K_{h}=15.3\times 10^{-6}cm/s$, while the initial permeability coefficient of soil filling was $K_{s}=7.2\times 10^{-4}cm/s$, in which case, $K_{s}=47K_{h}$ can be obtained. The permeability coefficient of the filling in the trunk grouting vein area decreased the most, compared with that before grouting, while the permeability coefficient of the permeable filling area decreased the least. It was concluded that, after splitting grouting, the generation of the grouting veins and the compaction of the grouting veins to the surrounding soil significantly reduced the permeability of the filling, which in turn increased the strength of the soil. The skeleton effect formed by the slurry veins was the main factor of performance improvement.

## 5. Discussion and Conclusions

Grouting technology is a changing and complex process, and model tests are an effective means to study the grouting reinforcement law and mechanism. This study introduced in detail the experimental process of simulated filling splitting grouting in the laboratory, and the grouting effect detection and evaluation methods were also provided. Tests of the soil filling performance after grouting were conducted. The following conclusions were drawn:

1. A model experiment system for simulating split grouting in filling soil was developed. The equipment could also meet the splitting grouting process of other soils and can be easily disassembled to support the observation and sampling analysis of the excavation of the grouting reinforcement body.
2. In the experiment, three types of grouting veins were formed by the split grouting of filling soil, namely, trunk grouting veins, branch grouting veins, and permeable filling grouting veins. The trunk grouting veins were centered on the grouting holes and distributed in a roughly horizontal plate shape. The intersecting branch grouting veins extended between the trunk grouting veins, and the penetration filling phenomenon occurred in the area away from the grouting hole and trunk grouting vein. The thickness of grouting veins near the grouting hole was larger and gradually decreased as it expanded further.
3. The filling soil was strengthened by the three-stage grouting vein network of trunk vein-branch, vein-permeation filling veins, and the compaction between soils, in which the trunk grouting veins contribute significantly towards strength improvement.
4. The results of the uniaxial compression test, dynamic preliminary test, and indoor geotechnical test showed that the equivalent compressive strength of filling increased by 186%; equivalent cohesion and internal friction angle increased by 45.3% and 44.9%, respectively; and equivalent permeability coefficient decreased by 47 times. The bearing capacity of the foundation was increased by 2–3 times.
5. The filling medium in this study was filling soil below the construction site. Owing to the split grouting in different types of media, the characteristics of the veins, reinforcement effect, and reinforcement mechanism were quite different. Thus, the conclusions obtained in this study could provide a solid reference for the design and theoretical research of grouting reinforcement in the filled soil layer and the detection of grouting effect. The applicability of other types of soil is still uncertain, and relevant research on other types of soil will be carried out in the future.

## Figures and Tables

**Table 1:** Basic physical-mechanical parameters of the soil.

Soil Type	Initial Water Content	$\mathrm{Natural}\;\mathrm{Density}/(g/cm^{3})$	Liquid Limit	Plastic Limit	Void Ratio
Filling	37.8%	1.814	44.2%	23.2%	0.32

**Table 2:** Results of compressive strength test.

	Modulus of Compression Before Grouting E /(MPa)	Modulus of Compression After Grouting E /(MPa)		
Sample Number	Injected Filling	Trunk Vein Area	Branch Vein Area	Permeable Filling Area
Sample 1	6.37	36.02	25.53	8.93
Sample 2	6.53	33.84	23.95	9.35
Sample 3	6.67	34.76	22.75	11.65
Sample 4	6.53	36.85	19.46	9.95
Sample 5	6.85	37.45	21.38	11.10
Sample 6	6.95	34.56	26.03	13.34
Average value	6.65	35.58	23.18	10.72

**Table 3:** Comparison of basic parameters of soil before and after grouting.

	Number of Samples	Density (g/cm^3^)	Water Content (%)	Average Cohesion (kPa)	Average Internal Friction Angle (°)	Cohesion Increases (%)	Increase in Internal Friction Angle (%)
Average before grouting	6	1.834	36.8	12.8	4.9	/	/
Trunk vein area	6	2.375	18.4	34.2	14.9	167	75.7
Branch vein area	6	1.910	28.7	19.5	6.8	52.3	38.8
Permeable filling area	6	1.856	34.4	14.4	5.9	12.5	20.4
Average after grouting	6	1.952	30.2	18.6	7.1	45.3	44.9

**Table 4:** Comparison of permeability coefficient of soil before and after grouting.

	Permeability Before Grouting/ $\left(10^{-4}cm/s\right)$	Permeability After Grouting/ $\left(10^{-6}cm/s\right)$		
Sample Number	Injected Filling	Trunk Vein Area	Branch Vein Area	Permeable Filling Area
Sample 1	8.5	0.23	0.9	26.3
Sample 2	7.0	0.07	3.5	31.6
Sample 3	5.3	0.11	2.8	44.2
Sample 4	8.0	0.09	1.4	34.8
Average value	7.2	0.12	2.2	34.2
